# Burden of silica-attributed pneumoconiosis and tracheal, bronchus & lung cancer for global and countries in the national program for the elimination of silicosis, 1990–2019: a comparative study

**DOI:** 10.1186/s12889-024-18086-9

**Published:** 2024-02-22

**Authors:** Lingfeng Min, Yiyang Mao, Hanpeng Lai

**Affiliations:** 1 Department of Respiratory and Critical Care Medicine, Northern Jiangsu People’s Hospital, 225009 Yangzhou, China; 2 Department of Occupational Health, Yangzhou Center for Disease Control and Prevention, 225001 Yangzhou, China; 3 Department of Occupational and Environmental Health, School of Public Health, Yangzhou University, 225009 Yangzhou, China

**Keywords:** Disease burden, Silica, Mortality, Disability-adjusted life year (DALY), Average annual percentage change (AAPC)

## Abstract

**Background:**

In industries worldwide, crystalline silica is pervasive and poses risks of pneumoconiosis and respiratory malignancies, with the latter being a knowledge gap in disease burden research that this study aims to address. By integrating both diseases, we also seek to provide an in-depth depiction of the silica-attributed disease burden.

**Methods:**

Data from the Global Burden of Disease 2019 were extracted to analyze the disease burden due to silica exposure. The trends of age-standardized mortality rate (ASMR) and age-standardized DALY rate (ASDR) from 1990 to 2019, as well as the age-specific number and rate of deaths and disability-adjusted life years (DALYs) in 1990 and 2019, were presented using GraphPad Prism software. The average annual percentage changes (AAPCs) on ASMR and ASDR were calculated using joinpoint regression models.

**Results:**

The global trends of disease burden due to silica exposure from 1990 to 2019 showed a significant decrease, with AAPCs on ASMR and ASDR of -1.22 (-1.38, -1.06) and − 1.18 (-1.30, -1.05), respectively. Vietnam was an exception with an unprecedented climb in ASMR and ASDR in general over the years. The age-specific deaths and DALYs mainly peaked in the age group 60–64. In comparison to 1990, the number of deaths and DALYs became higher after 45 years old in 2019, while their rates stayed consistently lower in 2019. Males experienced an elevated age-specific burden than females. China’s general age-standardized burden of pneumoconiosis and tracheal, bronchus & lung (TBL) cancer ranked at the forefront, along with the highest burden of pneumoconiosis in Chilean males and South African females, as well as the prominent burden of TBL cancer in Turkish males, Thai females, and overall Vietnamese. The age-specific burden of TBL cancer surpassed that of pneumoconiosis, and a delay was presented in the pneumoconiosis pinnacle burden compared to the TBL cancer. Besides, the burden of pneumoconiosis indicated a sluggish growth trend with advancing age.

**Conclusion:**

Our research highlights the cruciality of continuous enhancements in occupational health legislation for countries seriously suffering from industrial silica pollution and the necessity of prioritizing preventive measures for male workers and elderly retirees.

**Supplementary Information:**

The online version contains supplementary material available at 10.1186/s12889-024-18086-9.

## Background

Crystalline silica, being among the Earth’s most abundant minerals, serves as a crucial constituent of soil, sand, and granite [1]. Exposure to crystalline silica is omnipresent in the environment, encompassing not only natural phenomena like volcanic eruptions and sandstorms but also prevalent in numerous industries including ore mining, stone processing, fireproof materials manufacturing, and ceramic production. The estimated figures of workers exposed to industrial crystalline silica are 23 million in China [2], 11.5 million in India [3], 2 million in Europe [4], and 1.7 million in the United States [5]. In a multitude of socioeconomically challenged developing nations, the precise count of silica-exposed workers has been severely underestimated due to constraints in the existing monitoring approaches [6].

In recent times, the detrimental health impacts caused by silica exposure have garnered growing attention as a pressing public health problem [7]. Long-term inhalation of crystalline silica particles can directly lead to pneumoconiosis, an incurable respiratory occupational disease characterized by airway inflammation and interstitial fibrosis [8]. As of 2021, the global pneumoconiosis patient count stood at an estimation of 527,500 [9]. Out of these, 60,000 new cases were reported in 2017, marking an alarming 66% surge in comparison to the figures recorded in 1990 [10]. Since 2015, although there has been a slight decline in the prevalence of pneumoconiosis, the mortality associated remained persistently high, with a yearly death toll surpassing 21,000 [9]. To cope with the grave burden, nine countries including China have developed their own National Program for the Elimination of Silicosis (NPES) [11].

Tracheal, bronchus & lung cancer assumes a prominent position among occupational malignancies due to its strong correlation with silica exposure, whose carcinogenicity to human beings has received official confirmation from the International Agency for Research on Cancer (IARC) [12]. Upon considering adjustments for smoking and other hazard factors that contribute to tumor formation, previous studies have affirmed that prolonged occupational exposure to silica substantially enhances the mortality rate of lung cancer among workers, with a definitive dose-response relationship between the concentration of silica exposure and the magnitude of the excess mortality rate [13]. Over the past 30 years, occupational exposure to silica has emerged as a decisive factor contributing to lung cancer deaths in economically developing countries [14]. The fact remains that a considerable proportion of silica-expose workers also come into contact with other carcinogens, which are likely to synergize with silica to amplify their tumorigenic properties and thus pose a significant challenge to countries in their efforts to eradicate occupational respiratory malignant neoplasm [15].

Despite occasional literature reports on the disease burden of pneumoconiosis, there is a scarcity of targeted research specifically focusing on the burden of silica-attributed respiratory carcinomas. Therefore, our current study was dedicated to addressing this knowledge gap. Not only that, but our research would also employ the Global Burden of Disease (GBD) database to effectively integrate two diseases stemming from silica exposure, namely pneumoconiosis and respiratory cancer. To gain a thorough understanding, apart from delving into information on gender and age in depth, a particular emphasis would be placed on comparing the disparities in the burden of the two diseases that had been under widespread exploration among the nine countries attending the NPES program.

## Materials and methods

### Original data

Data utilized in our current study were acquired from GBD 2019 globally and among countries in the NPES, i.e., Brazil, Chile, China, India, Peru, South Africa, Thailand, Turkey, and Vietnam, from 1990 to 2019 (https://vizhub.healthdata.org/gbd-results/). The relevant information was extracted to analyze the disease burden attributable to silica exposure and the detailed instructions for extraction have been illustrated in the Additional File section. According to the hierarchy in GBD 2019, the “Risk” factor contained “Environmental/occupational risks”, “Behavioral risks”, and “Metabolic risks” at the primary level, with 20 secondary subtypes and 52 tertiary subtypes [16]. Under one of the tertiary risk factors, that is “Occupational carcinogens”, “Occupational exposure to silica” was selected in the “Risk” items. The corresponding tertiary causes, “Pneumoconiosis” (J60-J65) and “Tracheal, bronchus & lung (TBL) cancer” (C33-C34), were selected. They were situated within the secondary causes, categorized as “Neoplasms” and “Chronic respiratory diseases”, respectively, under the broader classification of “Non-communicable diseases” at the primary level. Both of them were identified as underlying causes of death according to the International Classification of Diseases, Tenth Edition (ICD-10) system.

### Statistical description

GBD 2019 employed age-standardized rates to characterize the disease burden due to silica exposure across regions, years, and genders. For generating the age-standardized rates, the World Health Organization (WHO) World Standard Population Distribution was used to allow comparisons among populations with different age structures [17]. The age-standardized rate of mortality and DALYs of causes from silica exposure between 1990 and 2019 were collected for global and nine countries in the NPES. The global number and rate of age-specific deaths and disability-adjusted life years (DALYs) attributable to silica exposure in 1990 and 2019 were extracted by gender. The methods for estimating DALYs attributable to risk factors were elucidated previously [18]. For further investigation of differences in disease burden related to silica exposure, the trend of age-standardized mortality rate (ASMR) and age-standardized DALY rate (ASDR) from 1990 to 2019, and the age-specific number and rate of deaths and DALYs in 1990 and 2019 were analyzed for two categories of causes due to silica exposure by gender. All the data presentation was conducted by using GraphPad Prism version 9.5 software (GraphPad Software Inc., CA, USA).

### Statistical inference

For the summarization of trends of ASMR and ASDR within a predetermined interval, the measures of average annual percentage changes (AAPCs) on ASMR and ASDR were calculated through the joinpoint regression analysis. The best-fitting points were identified by the changing slopes and connected to a series of log-linear models; with a maximum of 5 joint points [19]. The equations of joinpoint regression models were listed as follows:

$$ E\left(y|x\right)={e}^{{\beta }_{0}+{\beta }_{1}x+{\delta }_{1}\left(x-{{\tau }}_{1}\right)+\dots +{\delta }_{k}\left(x-{{\tau }}_{k}\right)+\dots }$$

where *k* was the number of turning points, *τ*_*k*_ was the unknown turning points, *β*_0_ was the constant, *β*_1_ was the regression coefficient, *δ*_*k*_ was the regression coefficient of the *k*th piecewise function. Therefore, AAPCs with their corresponding 95% confidence intervals (CIs) were obtained to quantify the overall trends, and only when the upper and lower limits of the CI were both < 0 or both > 0 would the AAPC be assessed to be significantly different from 0. The AAPC was estimated using Joinpoint version 5.0.2 software (National Cancer Institute, Bethesda, MD, USA).

## Results

### Overall trend of disease burden due to silica from 1990 to 2019 for global and NPES countries

**Fig. 1:**
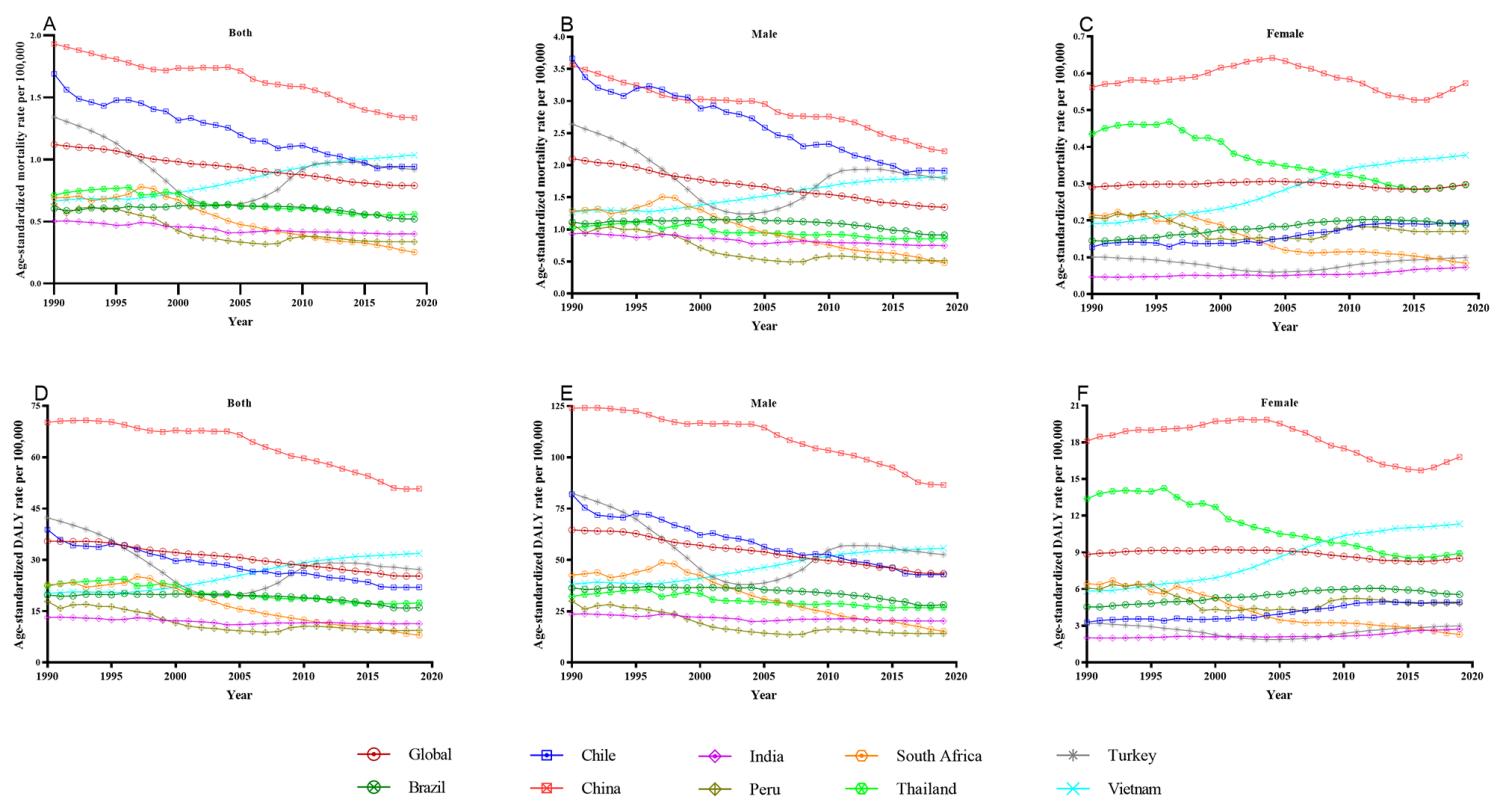
The age-standardized rates of aggregate mortality and DALY for pneumoconiosis and TBL cancer due to silica exposure for global, Brazil, Chile, China, India, Peru, South Africa, Thailand, Turkey, and Vietnam, from 1990 to 2019. DALY: disability-adjusted life year

The ASMR and ASDR of disease burden due to silica exposure showed an obvious downward trend on a global scale, especially among men, as exhibited in Fig. 1. China boasted a commanding lead over other countries in terms of silica-attributable disease burden except for ASMR in men. The ASMR and ASDR consistently decreased in the general population and males in China, where the declining tendency seemed not notable before 2005, but became more pronounced after that (Fig. 1A, B and D, and 1E). Despite some occasional fluctuations, a prevailing downward trend was evident in ASMR and ASDR in Chile and South Africa. Turkey’s ASMR and ASDR once dropped to their lowest point in 2003. Vietnam stood as a unique exception since its ASMR and ASDR were on a gradual increase year after year (Fig. 1A and D). The national silicosis elimination programs mainly began in the first decade of this century for most countries, coinciding with a notably accelerated decline in ASMR and ASDR observed in Chilean male workers (Fig. 1B and E), Thai female workers (Fig. 1C and F), and general South African workers (Fig. 1A and D). More original data were displayed in Tables S1 and S2.

As offered in Table 1, the global disease burden due to silica exposure from 1990 to 2019 exhibited a significant annual change rate, with AAPCs on ASMR and ASDR in the general population of -1.22 (-1.38, -1.06) and − 1.18 (-1.30, -1.05), respectively. The largest annual rate of change was observed in South Africa, reaching − 3.45 (-3.92, -2.98) and − 3.58 (-4.11, -3.04) on ASMR and ASDR in general, respectively. The annual change rate in Vietnam, on the contrary, displayed an unprecedented surge, as evidenced by AAPCs on ASMR and ASDR of 1.54 (1.45, 1.63) and 1.58 (1.51, 1.66), respectively in general. Females in Brazil, Chile, and India also experienced anomalous growth.

**Table 1:** Overall trends on age-standardized rates o mortality and DALY from silica exposure, 1990–2019

Country	AAPCs on ASMR from 1990 to 2019			AAPCs on ASDR from 1990 to 2019		
	Both Sexes	Male	Female	Both Sexes	Male	Female
Global	-1.22 (-1.38, -1.06)	-1.53 (-1.72, -1.34)	0.08 (-0.07, 0.22)	-1.18 (-1.30, -1.05)	-1.38 (-1.54, -1.22)	-0.13 (-0.25, -0.01)
Brazil	-0.51 (-0.64, -0.37)	-0.70 (-0.85, -0.55)	0.98 (0.88, 1.08)	-0.77 (-0.93, -0.62)	-0.89 (-1.16, -0.61)	0.73 (0.56, 0.90)
Chile	-2.14 (-2.58, -1.69)	-2.38 (-2.83, -1.92)	1.37 (0.86, 1.88)	-1.96 (-2.53, -1.39)	-2.28 (-2.84, -1.71)	1.37 (1.06, 1.70)
China	-1.29 (-1.44, -1.14)	-1.67 (-1.85, -1.48)	0.05 (-0.09, 0.19)	-1.17 (-1.36, -0.98)	-1.30 (-1.59, -1.01)	-0.27 (-0.46, -0.08)
India	-0.82 (-1.30, -0.33)	-0.78 (-1.29, -0.27)	1.54 (1.12, 1.98)	-0.52 (-0.95, -0.08)	-0.54 (-0.86, -0.22)	1.04 (0.79, 1.28)
Peru	-2.17 (-3.20, -1.13)	-2.53 (-3.89, -1.16)	-0.73 (-1.31, -0.14)	-2.17 (-3.13, -1.20)	-2.55 (-3.83, -1.25)	-0.84 (-1.45, -0.23)
South Africa	-3.45 (-3.92, -2.98)	-3.40 (-3.92, -2.88)	-3.18 (-3.70, -2.66)	-3.58 (-4.11, -3.04)	-3.54 (-4.23, -2.84)	-3.57 (-4.11, -3.03)
Thailand	-0.96 (-1.30, -0.62)	-0.67 (-1.06, -0.27)	-1.39 (-1.75, -1.03)	-0.99 (-1.32, -0.68)	-0.71 (-1.06, -0.35)	-1.49 (-1.78, -1.19)
Turkey	-1.28 (-1.58, -0.98)	-1.30 (-1.60, -0.99)	-0.05 (-0.35, 0.25)	-1.51 (-1.82, -1.19)	-1.54 (-1.81, -1.26)	-0.23 (-0.54, 0.08)
Vietnam	1.54 (1.45, 1.63)	1.26 (1.16, 1.36)	2.37 (2.24, 2.51)	1.58 (1.51, 1.66)	1.32 (1.22, 1.41)	2.33 (2.13, 2.53)

DALY: disability-adjusted life year; AAPC: average annual percentage change; ASMR: age-standardized mortality rate; ASDR: age-standardized DALY rate

### Overall age-specific disease burden due to silica in 1990 and in 2019

**Fig. 2:**
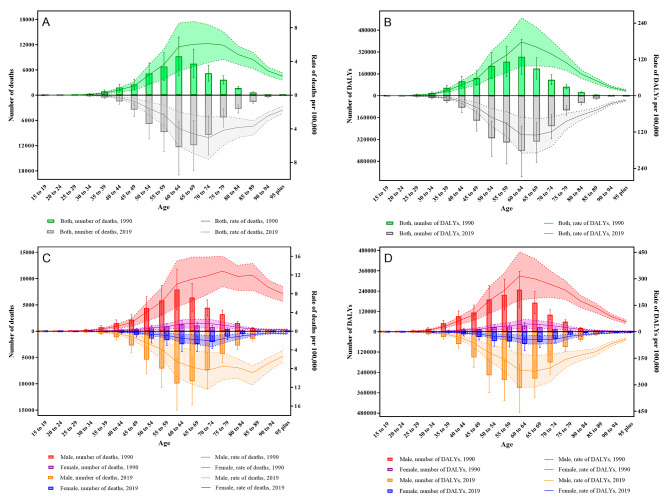
The global age-specific numbers and rates of aggregate mortality and DALY for pneumoconiosis and TBL cancer due to silica exposure in 1990 and in 2019. DALY: disability-adjusted life year

As displayed in Fig. 2A, the worldwide death cases from silica exposure exhibited a noticeable increase starting at the age of 30, with the age-specific deceased numbers reaching the peak point in the age group 60–64, and the age-specific mortality achieving the zenith in the age group 70–74 in both 1990 and 2019. Compared to 1990, the death number before the age of 45 remained relatively lower and a reversal appeared after 45 years old when the death number became higher in 2019. However, the mortality stayed consistently lower in 2019 than that in 1990 across all age groups. In Fig. 2B, the global silica-attributable DALY demonstrated a similar growth trend from the age of 25, where the age-specific DALY numbers and DALY rates both hit their highest point in the age group 60–64 in both 1990 and 2019; despite that, the subsequent DALYs decreased at a more rapid pace. The global age-specific death and DALY due to silica exposure for males were elevated compared to females across various age ranges in both 1990 and 2019 as revealed in Fig. 2C and D. The trend of mortality and DALY with increasing age among men and women was almost akin to the general population. More original data were displayed in Table S3.

### Trend of cause-specific disease burden due to silica from 1990 to 2019 for global and NPES countries

**Fig. 3:**
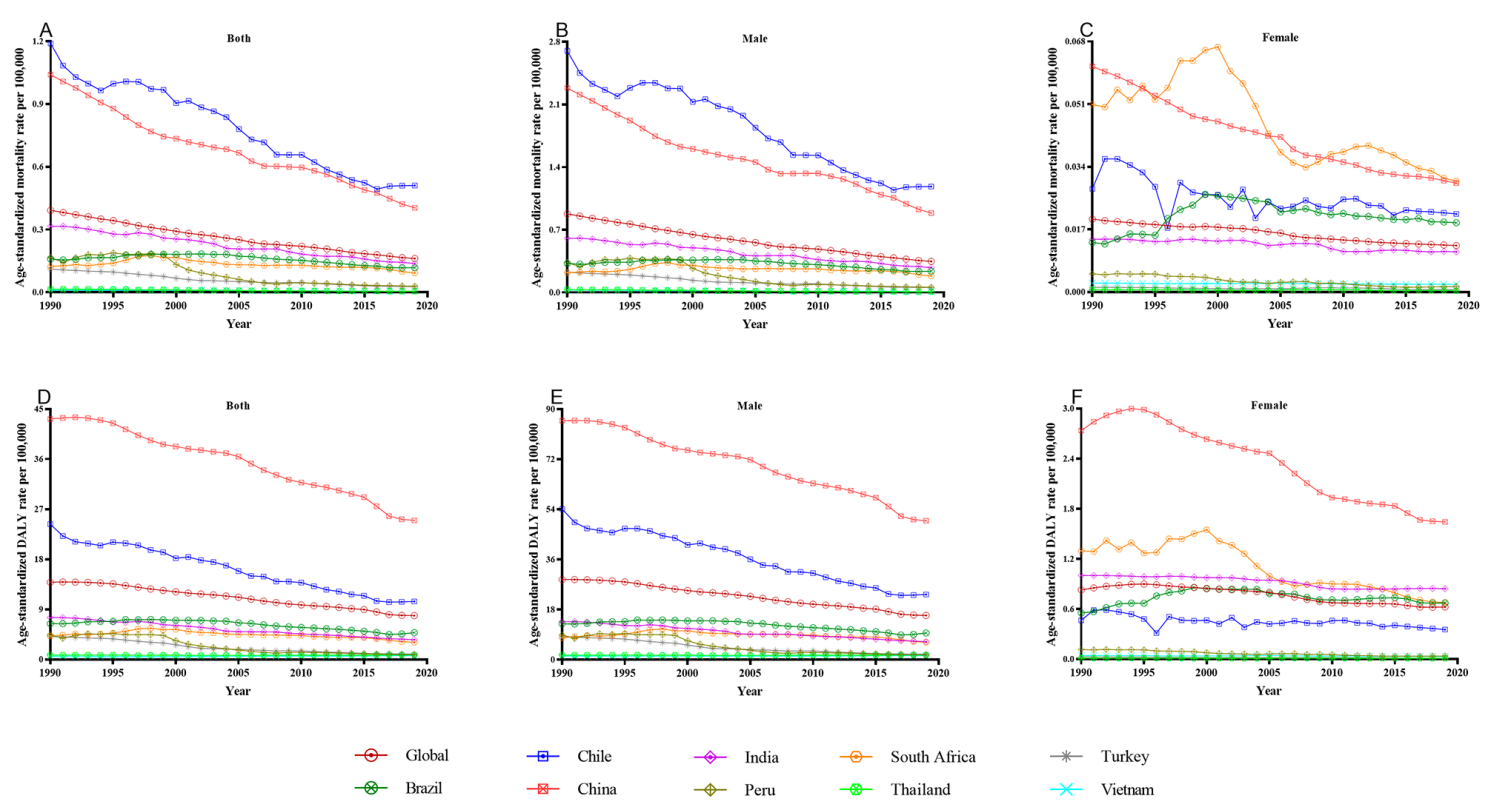
The age-standardized rates of pneumoconiosis mortality and DALY due to silica exposure for global, Brazil, Chile, China, India, Peru, South Africa, Thailand, Turkey, and Vietnam, from 1990 to 2019. DALY: disability-adjusted life year

**Fig. 4:**
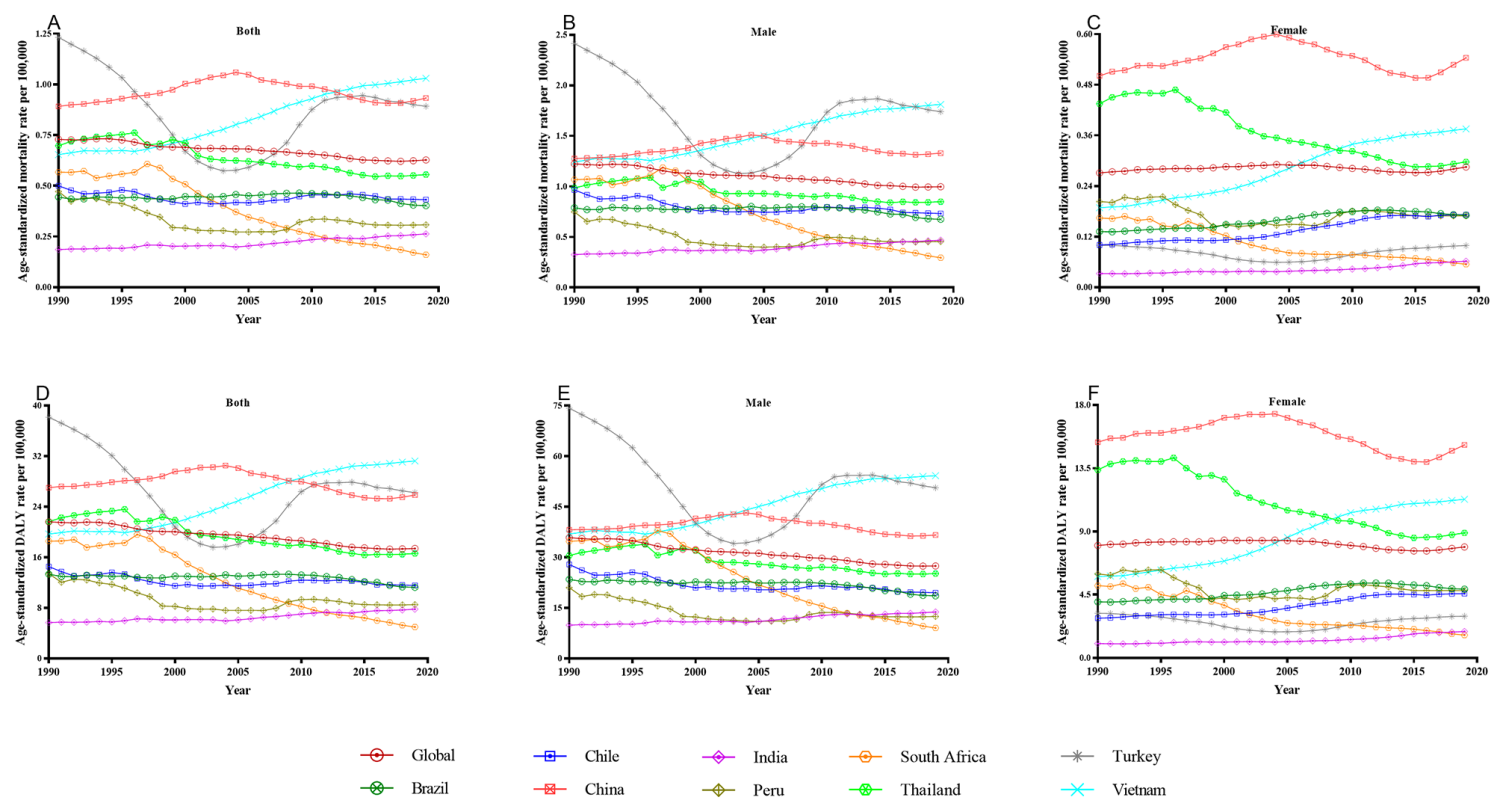
The age-standardized rates of TBL cancer mortality and DALY due to silica exposure for global, Brazil, Chile, China, India, Peru, South Africa, Thailand, Turkey, and Vietnam, from 1990 to 2019. DALY: disability-adjusted life year; TBL: tracheal, bronchus & lung

The ASMR and ASDR of TBL cancer and pneumoconiosis demonstrated a striking downward trend at a global level, with a specific focus on males, as presented in Figs. 3 and 4. China and Chile both shared a noticeably higher ASMR of pneumoconiosis in the general population and males (Fig. 3A and B), while South Africa was another high-burden nation for female pneumoconiosis alongside China (Fig. 3C). As for TBL cancer, the ASMR and ASDR rankings of Turkey, China, and Vietnam in the general population and males were among the highest (Fig. 4A, B, D and E); Turkey, initially experiencing an absolute advantage, underwent a swift decline until 2003, whereas Vietnam experienced a rapid rise in the recent decade. China, in addition, took the lead in the ASMR and ASDR of TBL cancer among women, with Thailand and Vietnam following closely behind (Fig. 4C and F). In the first decade of the 21st century, with the widespread implementation of silica elimination programs, there was a remarkable decrease in the burden of pneumoconiosis for Chinese general workers (Fig. 3A and D), Chilean male workers (Fig. 3B and E), and South African female workers (Fig. 3C and F), as well as a substantial reduction in the burden of TBL cancer for general South African workers (Fig. 4A and D), Turkish male workers (Fig. 4B and E), and Thai female workers (Fig. 4C and F). More original data were displayed in Tables S4, S5, S6 and S7.

As depicted in Table 2, the global burden of pneumoconiosis and TBL cancer from 1990 to 2019 demonstrated a significant annual change rate in the general population, as evidenced by the AAPCs of pneumoconiosis on ASMR and ASDR of -3.05 (-3.22, -2.88) and − 1.96 (-2.21, -1.72), respectively, and AAPCs of TBL cancer on ASMR and ASDR of -0.52 (-0.61, -0.42) and − 0.74 (-0.84, -0.65), respectively. Pneumoconiosis among almost all NPES countries showed a reduced rate of annual change. However, the TBL cancer burden in India and Vietnam underwent unprecedented growth, while both Brazil and Chile also experienced similarly atypical upward rates of annual change in the burden of TBL cancer among women.

**Table 2:** Trends on age-standardized rates of cause-specific mortality and DALY from silica exposure, 1990–2019

Country	AAPCs on ASMR from 1990 to 2019			AAPCs on ASDR from 1990 to 2019		
	Both Sexes	Male	Female	Both Sexes	Male	Female
Pneumoconiosis						
Global	-3.05 (-3.22, -2.88)	-3.18 (-3.36, -3.00)	-1.53 (-1.73, -1.32)	-1.96 (-2.21, -1.72)	-2.05 (-2.29, -1.81)	-0.99 (-1.09, -0.90)
Brazil	-1.03 (-1.37, -0.70)	-1.08 (-1.35, -0.80)	1.13 (0.53, 1.74)	-0.99 (-1.41, -0.56)	-1.01 (-1.42, -0.59)	0.63 (-0.03, 1.30)
Chile	-2.85 (-3.51, -2.20)	-2.73 (-3.39, -2.06)	-1.40 (-2.03, -0.77)	-2.99 (-3.43, -2.55)	-2.96 (-3.41, -2.51)	-1.05 (-1.57, -0.53)
China	-3.23 (-3.42, -3.04)	-3.20 (-3.40, -3.01)	-2.50 (-2.74, -2.25)	-1.96 (-2.36, -1.55)	-1.94 (-2.33, -1.54)	-1.83 (-2.19, -1.46)
India	-2.82 (-3.09, -2.56)	-2.65 (-3.46, -1.84)	-0.87 (-1.71, -0.02)	-2.57 (-3.04, -2.09)	-2.59 (-3.16, -2.02)	-0.59 (-0.70, -0.47)
Peru	-5.66 (-6.52, -4.79)	-5.66 (-6.53, -4.78)	-4.06 (-5.72, -2.37)	-5.29 (-6.07, -4.50)	-5.27 (-6.06, -4.48)	-3.72 (-5.14, -2.28)
South Africa	-1.04 (-1.59, -0.49)	-0.66 (-1.65, 0.33)	-1.68 (-2.41, -0.94)	-1.14 (-1.79, -0.48)	-0.87 (-1.77, 0.03)	-2.39 (-3.32, -1.44)
Thailand	-4.30 (-4.49, -4.10)	-4.49 (-4.69, -4.29)	-1.54 (-2.60, -0.48)	-0.09 (-0.26, 0.08)	-0.04 (-0.20, 0.13)	-1.15 (-2.19, -0.10)
Turkey	-4.78 (-5.23, -4.32)	-4.74 (-5.18, -4.29)	-2.26 (-2.83, -1.68)	-4.86 (-5.60, -4.12)	-4.86 (-5.56, -4.15)	-1.99 (-2.30, -1.68)
Vietnam	-2.39 (-2.43, -2.36)	-2.87 (-2.90, -2.83)	-0.56 (-0.62, -0.49)	0.83 (0.76, 0.90)	0.74 (0.66, 0.81)	-0.73 (-0.79, -0.67)
Tracheal, Bronchus, and Lung Cancer						
Global	-0.52 (-0.61, -0.42)	-0.72 (-0.87, -0.56)	0.17 (0.03, 0.32)	-0.74 (-0.84, -0.65)	-0.92 (-1.07, -0.77)	-0.05 (-0.16, 0.07)
Brazil	-0.33 (-0.46, -0.21)	-0.51 (-0.60, -0.41)	0.93 (0.81, 1.05)	-0.58 (-0.70, -0.45)	-0.77 (-0.87, -0.67)	0.74 (0.62, 0.85)
Chile	-0.52 (-0.96, -0.07)	-0.97 (-1.45, -0.48)	1.87 (1.55, 2.18)	-0.80 (-1.25, -0.36)	-1.24 (-1.77, -0.71)	1.66 (1.37, 1.95)
China	0.15 (-0.03, 0.34)	0.14 (-0.11, 0.39)	0.26 (0.12, 0.41)	-0.15 (-0.38, 0.07)	-0.14 (-0.33, 0.05)	-0.08 (-0.19, 0.03)
India	1.17 (0.84, 1.50)	1.25 (1.08, 1.42)	2.24 (1.88, 2.59)	1.12 (0.82, 1.42)	1.15 (0.98, 1.32)	2.15 (1.70, 2.61)
Peru	-1.46 (-2.33, -0.58)	-1.66 (-2.67, -0.65)	-0.67 (-1.24, -0.10)	-1.56 (-2.46, -0.67)	-1.76 (-2.78, -0.73)	-0.80 (-1.41, -0.20)
South Africa	-4.23 (-4.76, -3.70)	-4.38 (-4.91, -3.85)	-3.77 (-4.31, -3.24)	-4.45 (-4.95, -3.95)	-4.59 (-5.17, -4.01)	-3.96 (-4.55, -3.38)
Thailand	-0.91 (-1.25, -0.57)	-0.59 (-0.99, -0.20)	-1.37 (-1.70, -1.05)	-1.04 (-1.37, -0.72)	-0.75 (-1.11, -0.39)	-1.49 (-1.78, -1.19)
Turkey	-1.09 (-1.39, -0.78)	-1.10 (-1.40, -0.80)	-0.01 (-0.30, 0.28)	-1.27 (-1.66, -0.88)	-1.30 (-1.58, -1.03)	-0.22 (-0.54, 0.10)
Vietnam	1.58 (1.49, 1.67)	1.31 (1.21, 1.41)	2.40 (2.26, 2.53)	1.60 (1.52, 1.68)	1.34 (1.24, 1.43)	2.35 (2.15, 2.55)

DALY: disability-adjusted life year; AAPC: average annual percentage change; ASMR: age-standardized mortality rate; ASDR: age-standardized DALY rate

### Age-specific cause-specific disease burden due to silica in 1990 and in 2019

**Fig. 5:**
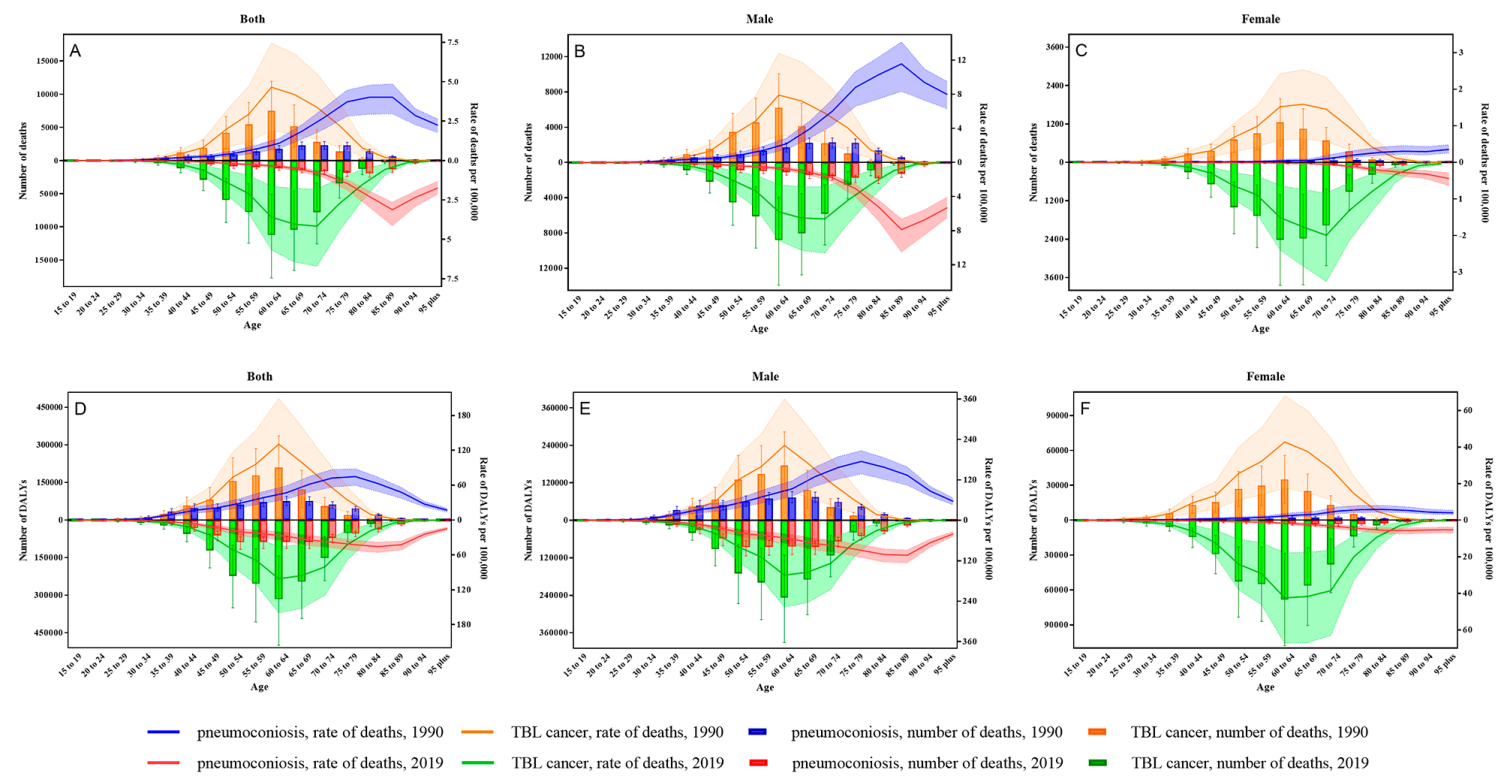
The global age-specific numbers and rates of cause-specific (pneumoconiosis and TBL cancer) death and DALY due to silica exposure in 1990 and in 2019. DALY: disability-adjusted life year; TBL: tracheal, bronchus & lung

According to Fig. 5A and D, no matter in 1990 or 2019, when excluding certain young adults and the elderly, the worldwide disease burden of TBL cancer due to silica exposure surpassed that of pneumoconiosis. The maximum of TBL cancer deaths and DALYs was predominantly observed at the age of 60–64. In contrast to 1990, the year 2019 witnessed an upsurge in both the death and DALY number of TBL cancer across all age brackets, while the mortality and DALY rates experienced a noticeable rise after the age of 70. The pinnacle disease burden of pneumoconiosis manifested itself after 65 years old, presenting a delay compared to the zenith period of TBL cancer. Even though the pneumoconiosis death number in the 80 + age group, as well as the DALY number in the 45 + age group, exceeded the levels in 1990, the mortality and DALY rates in every age cohort stayed lower than that recorded in 1990. The men’s burden of two specific causes demonstrated a trend resembling that of the general population as age increased, as illustrated in Fig. 5B and E. Based on Fig. 5C and F, it was observed that the disease burden of TBL cancer overwhelmingly exceeded that of pneumoconiosis in female workers, although the disease burden of pneumoconiosis indicated an extremely sluggish growth trend with advancing age. More original data were displayed in Tables S8 and S9.

## Discussion

The current study reported the recent trends in deaths and DALYs of silica-attributed disease burden with a focus on age, gender, cause, and regional divisions, over the past three decades. Based on the GBD 2019 data, although a downward trend in mortality and DALY due to silica exposure existed at a global level, the situation seemed not to be entirely consistent in countries suffering from long-term and serious industrial silica pollution. The silica-induced disease burden was concentrated in the elderly population, with higher mortality and DALY found in males for all age groups and in TBL cancer patients for most age groups.

During the past 30 years, substantial improvement has been achieved globally in the containment of silica, as our research revealed, while the burden of diseases associated with silica exposure has shown a substantial reduction. Unfortunately, the changes in the burden of diseases among female workers displayed a lack of consistency to the worldwide trend, while the women’s annual rate of change on the ASMR did not show a significant pattern, potentially attributed to the unusually positive AAPCs in female workers among several populous countries, e.g., Brazil, China, and India. Additionally, the female workers’ annual rate of change on ASMR for silica-attributed TBL cancer also demonstrated a weak growing tendency, prompting us to be aware of the probable consequences caused by occupational carcinoma burden in females on socio-economic development. Our results showed that both ASMR and ASDR in women correspond to 25% of the men, but this was seriously inconsistent with the actual proportion of male and female industrial workers in most countries. For example, in Brazil and Chile, male workers have occupied the vast majority of mining and mineral transformation jobs. A reasonable explanation for the women’s increasing burden of TBL cancer was the rapid growth in tobacco product consumption among female workers in more recent decades..

Inconsistency was also observed in the annual trend of disease burden among countries encountering serious occupational silica pollution, which signified the discrepancies in economic progress and occupational health regulations among nations [20]. China occupied the top spot among NPES countries regarding the disease burden associated with silica exposure, and the accelerated decrease of its disease burden, particularly pneumoconiosis after 2005 could be credited to systematic industrial upgrading with the widespread introduction of ventilation equipment and wet operation methods [21]. Chilean male workers also exhibit persistently high ASMR of pneumoconiosis, which could be largely blamed on their extensive reliance on the copper mining industry [22]. South Africa, known for its prominent gold mining industry, had a notable involvement of women in platinum transportation and processing, accounting for the alarmingly high ASMR of pneumoconiosis among female workers which occasionally surpassed that of China [23]. The flourishing growth of construction materials production, automobile manufacturing, as well as steel and non-ferrous metal processing industries, contributed to Turkey’s overwhelming burden of TBL cancer in the early years [24]. Fortunately, the Turkish government had been committed to occupational hygiene legislation and the disease burden was largely brought under control, despite a temporary rebound in 2003 arising from the inadequate implementation of protective measures accompanying new equipment [25]. In the last 30 years, Vietnam has faced a gradual and continuous climb in the disease burden due to silica exposure, with TBL cancer being a critical concern, as suggested by the positive AAPCs, which presented substantial challenges to the country’s initiatives in industrial silica control [26]. The upward trends in Vietnam possibly originated from the implementation of the NPES program involving training and improved diagnosis of older workers who had been previously unrecognized with silica-related diseases. The remaining countries, e.g. Brazil, showed only a very slight downward trend in the burden of silica-related diseases, possibly due to variations in the incidence across different industries and age groups domestically [27].

Within the two specific causes due to silica exposure, TBL cancer occupied a much greater proportion, mainly on account of the carcinogenicity of silica that had been officially recognized [12]. On one side, silica could directly trigger the onset of tumorigenesis by causing DNA damage and mutations in respiratory epithelial cells; on the flip side, gradual malignant transformation would occur in pulmonary fibrous nodules as a result of sustained inflammatory stimulation [28]. TBL cancer is strongly associated with the presence of silicosis; hence, typically, the mortality curves of the two should exhibit similarity. However, there was a significant discrepancy between the DALYs for pneumoconiosis and TBL cancer from Turkey. This could be explained by a considerable portion of workers exposed to a variety of occupational risk factors simultaneously, whose potential causes for occupational TBL cancer were specified as factors other than silica. The elusive presentation of early signs of TBL cancer also made the screening process more complex, resulting in delayed diagnosis and missed opportunities for the optimal treatment window [29]. Therefore, it is crucial to offer early screening technologies for occupational populations at high risk of lung cancer from exposure to silica or other industrial pollutants. Commonly adopted techniques, for example, low-dose computed tomography (CT), have been proven to significantly enhance the accuracy of diagnosing early malignant lung nodules [30]. In addition, it is worth noting that the peak age range for the disease burden of TBL cancer appeared earlier than that of pneumoconiosis, as the development of malignant neoplasm was also affected by a combination of factors including cigarette smoking, radiation operation, and industrial pollutants, undoubtedly reducing the availability of the labor force and increasing extra social security expenditures [31].

The disease burden showed considerable distinctions between the two genders. With the progression of age, males encountered a notable increase and subsequent decrease in the burden of pneumoconiosis, suggesting the dominance of males in traditional industries with the production of silica exposure at high levels, for example, ore mining, stone cutting, and glass processing [32]. The peak burden of male pneumoconiosis emerging after 65 years old implied that its impact on employees was more reflected in their personal quality of life during their senior years rather than their labor productivity; nonetheless, it is crucial to guarantee the provision of regular medical surveillance even after their retirement [33]. Females, conversely speaking, exhibited an uncommonly slow-paced rise in pneumoconiosis mortality and DALY as age advanced, highlighting the fact that female employees, despite being exposed to occupational silica dust, tended to be clustered in industries with relatively lower levels of pollution [34]. In recent years, an increasing number of women from developing nations have set out to join the workforce, further augmenting their potential for silica exposure [35].

The age range reaching the maximum disease burden due to silica exposure in 2019 was generally delayed than that in 1990, underscoring the efforts made to control silica had brought about a certain degree of age postponement in the onset of associated diseases, while suggesting the mounting challenge of implementing relevant social security measures in the context of an aging population [36]. Additional evidence of the intensifying situation regarding silica oversight was the actual number of deaths and DALYs in the elderly in 2019 which still exceeded those of 1990, although the mortality and DALY rates in 2019 were lower across the board compared to 1990.

Before outlining our conclusions, we must confront the unavoidable shortcomings that exist in the current study. First of all, the data utilized was sourced from GBD 2019 which was gathered from multiple avenues, and the unevenness in data quality has impacted the robustness of our findings. Secondly, the GBD 2019 did not collect other silica-related diseases apart from TBL cancer and pneumoconiosis, and unfortunately, some patients’ direct causes of death had been classified as other respiratory diseases or malignant neoplasms, potentially leading to an underestimation of the real disease burden due to silica exposure. At last, given that our data was estimated from monitoring systems, there were probably temporal lags in different metrics that accurately represented the actual burden.

## Conclusion

Our research has pointed out the unfavorable situation for occupational silica control in several countries heavily affected by serious industrial silica pollution despite an overall downward trend in global silica-attributed disease burden, and it is thus imperative to continue advancing occupational health legislation there. In light of the higher burden of silica-related diseases observed in male workers, elderly retirees, and TBL cancer patients, underscores the necessity of prioritizing these three specific cohorts when enacting preventive measures for addressing occupational silica hazards.

### Electronic supplementary material

Below is the link to the electronic supplementary material.

Supplementary Material 1

Supplementary Material 2

Supplementary Material 3

Supplementary Material 4

Supplementary Material 5

Supplementary Material 6

Supplementary Material 7

Supplementary Material 8

Supplementary Material 9

Supplementary Material 10

## Data Availability

The datasets generated and analyzed during the current study are available in the Global Burden of Disease 2019 datasets (https://vizhub.healthdata.org/gbd-results/) Not applicable.

## Abbreviations

AAPC: Average annual percentage change
ASDR: Age-standardized DALY rate
ASMR: Age-standardized mortality rate
CI: Confidence interval
CT: Computed tomography
DALY: Disability-adjusted life year
GBD: Global Burden of Disease
IARC: International Agency for Research on Cancer
ICD-10: International Classification of Diseases, Tenth Edition
NPES: National program for the elimination of silicosis
TBL: Tracheal, bronchus & lung
WHO: World Health Organization

## References

[1] Tolstoy A, Lesovik V, Fediuk R, Amran M, Gunasekaran M, Vatin N, Vasilev Y. Production of greener high-strength concrete using Russian quartz sandstone mine waste aggregates. Materials. 2020;13(23). 10.3390/ma13235575 PMC7730853 33297576

[2] Wang D, Yang M, Liu Y, Ma J, Shi T, Chen W (2020). Association of silica dust exposure and cigarette smoking with mortality among mine and pottery workers in China. JAMA Netw Open.

[3] Rupani MP (2023). Challenges and opportunities for silicosis prevention and control: need for a national health program on silicosis in India. J Occup Med Toxicol.

[4] Maciejewska A (2008). Occupational exposure assessment to crystalline silica dust: Approach in Poland and worldwide. Int J Occup Med Environ Health.

[5] Hoy RF, Chambers DC (2020). Silica-related diseases in the modern world. Allergy.

[6] Gottesfeld P, Andrew D, Dalhoff J (2015). Silica exposures in artisanal small-scale gold mining in Tanzania and implications for tuberculosis prevention. J Occup Environ Hyg.

[7] Maciejewska A (2015). Health effects of occupational exposure to crystalline silica in the light of current research results. Med Pracy.

[8] Zhang N, Liu K, Wang K, Zhou C, Wang H, Che S, Liu Z, Yang H (2019). Dust induces lung fibrosis through dysregulated DNA methylation. Environ Toxicol.

[9] Qi X-M, Luo Y, Song M-Y, Liu Y, Shu T, Liu Y, Pang J-L, Wang J, Wang C (2021). Pneumoconiosis: current status and future prospects. Chin Med J.

[10] Shi P, Xing X, Xi S, Jing H, Yuan J, Fu Z, Zhao H (2020). Trends in global, regional and national incidence of pneumoconiosis caused by different aetiologies: an analysis from the global burden of Disease Study 2017. Occup Environ Med.

[11] Fedotov I. The ILO/WHO global programme for the elimination of silicosis. Occupational Health Southern. Africa 2006:4.

[12] Humans, IWGotEoCRt. Silica dust, crystalline, in the form of quartz or cristobalite. Arsenic, metals, Fibres and dusts. edn.: International Agency for Research on Cancer; 2012.

[13] Liu Y, Steenland K, Rong Y, Hnizdo E, Huang X, Zhang H, Shi T, Sun Y, Wu T, Chen W (2013). Exposure-response analysis and risk assessment for lung cancer in relationship to silica exposure: a 44-year cohort study of 34,018 workers. Am J Epidemiol.

[14] Zhang Y, Mi M, Zhu N, Yuan Z, Ding Y, Zhao Y, Lu Y, Weng S, Yuan Y (2023). Global burden of tracheal, bronchus, and lung cancer attributable to occupational carcinogens in 204 countries and territories, from 1990 to 2019: results from the global burden of disease study 2019. Ann Med.

[15] Lai H, Liu Y, Zhou M, Shi T, Zhou Y, Weng S, Chen W (2018). Combined effect of silica dust exposure and cigarette smoking on total and cause-specific mortality in iron miners: a cohort study. Environ Health.

[16] Murray CJ, Aravkin AY, Zheng P, Abbafati C, Abbas KM, Abbasi-Kangevari M, Abd-Allah F, Abdelalim A, Abdollahi M, Abdollahpour I (2020). Global burden of 87 risk factors in 204 countries and territories, 1990–2019: a systematic analysis for the global burden of Disease Study 2019. Lancet.

[17] Chen S, Liu M, Xie F (2022). Global and national burden and trends of mortality and disability-adjusted life years for silicosis, from 1990 to 2019: results from the Global Burden of Disease study 2019. BMC Pulm Med.

[18] Devleesschauwer B, Havelaar AH, Maertens de Noordhout C, Haagsma JA, Praet N, Dorny P, Duchateau L, Torgerson PR, Van Oyen H, Speybroeck N (2014). Calculating disability-adjusted life years to quantify burden of disease. Int J Public Health.

[19] Clegg LX, Hankey BF, Tiwari R, Feuer EJ, Edwards BK (2009). Estimating average annual per cent change in trend analysis. Stat Med.

[20] Ncube F, Kanda A (2018). Current status and the future of occupational safety and health legislation in low-and middle-income countries. Saf Health work.

[21] Li CZ, Zhao Y, Xu X (2019). Investigation of dust exposure and control practices in the construction industry: implications for cleaner production. J Clean Prod.

[22] Akchurin M (2020). Mining and defensive mobilization: explaining opposition to extractive industries in Chile. Sociol Dev.

[23] Knight D, Ehrlich R, Cois A, Fielding K, Grant AD, Churchyard G (2020). Predictors of silicosis and variation in prevalence across mines among employed gold miners in South Africa. BMC Public Health.

[24] Cangır AK, Yumuk PF, Sak SD, Akyürek S, Eralp Y, Yılmaz Ü, Selek U, Eroğlu A, Tatlı AM, Dinçbaş FÖ. Lung cancer in Turkey. In., Elsevier; 2022;17:1158–1170. 10.1016/j.jtho.2022.06.001 36192076

[25] Şen S, Barlas G, Yakıştıran S, Derin İG, Şerifi BA, Özlü A, Braeckman L, van der Laan G, van Dijk F (2019). Prevention of occupational diseases in Turkey: deriving lessons from journey of surveillance. Saf Health work.

[26] Lan TN, Son PH, van Trung L, Tu NTH, Keifer M, Barnhart S (2003). Distribution of silica-exposed workers by province and industry in Vietnam. Int J Occup Environ Health.

[27] Algranti E, Saito CA, Carneiro AP, Bussacos MA (2021). Mortality from silicosis in Brazil: temporal trends in the period 1980–2017. Am J Ind Med.

[28] Borm PJ, Tran L, Donaldson K (2011). The carcinogenic action of crystalline silica: a review of the evidence supporting secondary inflammation-driven genotoxicity as a principal mechanism. Crit Rev Toxicol.

[29] Birring S, Peake M. Symptoms and the early diagnosis of lung cancer. In., BMJ Publishing Group Ltd; 2005;60:268–269. 10.1136/thx.2004.032698 PMC1747375 15790977

[30] Yankelevitz DF, Yip R, Smith JP, Liang M, Liu Y, Xu DM, Salvatore MM, Wolf AS, Flores RM, Henschke CI (2015). CT screening for lung cancer: nonsolid nodules in baseline and annual repeat rounds. Radiology.

[31] Hirsch FR, Scagliotti GV, Mulshine JL, Kwon R, Curran WJ, Wu Y-L, Paz-Ares L (2017). Lung cancer: current therapies and new targeted treatments. Lancet.

[32] Chen WH, Liu YW, Wang HJ, Hnizdo E, Sun Y, Su LP, Zhang XK, Weng SF, Bochmann F, Hearl FJ et al. Long-term exposure to silica dust and risk of total and cause-specific mortality in Chinese workers: a cohort study. PLoS Med. 2012;9(4). 10.1371/journal.pmed.1001206 PMC3328438 22529751

[33] Barber CM, Fishwick D, Carder M, Van Tongeren M (2019). Epidemiology of silicosis: reports from the SWORD scheme in the UK from 1996 to 2017. Occup Environ Med.

[34] Poinen-Rughooputh S, Rughooputh MS, Guo Y, Lai H, Sun W, Chen W (2021). Sex-related differences in the risk of silicosis among Chinese pottery workers: a cohort study. J Occup Environ Med.

[35] Wai Y, Tarlo S (2003). Occupational lung disease in women. Eur Respir Monograph.

[36] Lukyanets A, Okhrimenko I, Egorova M (2021). Population aging and its impact on the country’s economy. Soc Sci Q.

