# Supplementary material for: Burden of silica-attributed pneumoconiosis and tracheal, bronchus & lung cancer for global and countries in the national program for the elimination of silicosis, 1990–2019: a comparative study
Source: BMC Public Health. 2024 Feb 22;24:571. doi: 10.1186/s12889-024-18086-9 (PMC10885413; doi:10.1186/s12889-024-18086-9)
Supplement: Supplementary file 1 — Supplementary Material 1 [file 12889_2024_18086_MOESM1_ESM.docx]

**Detailed Process of Extraction**

To accurately extract the target data, "Risk factor" should be selected in the "GBD Estimate" column; "Deaths" and "DALYs" should be selected in the "Measure" column; "Number" and "Rate" should be selected in the "Metric" column; "Global", "Brazil”, “Chile”, “China”, “India”, “Peru”, “South Africa”, “Thailand”, “Turkey” and “Vietnam” should be selected in the "Location" column; “15 to 19”, “20 to 24”, “25 to 29”, “30 to 34”, “35 to 39”, “40 to 44”, “45 to 49”, “50 to 54”, “55 to 59”, “60 to 64”, “65 to 69”, “70 to 74”, “75 to 79”, “80 to 84”, “85 to 89”, “90 to 94”, “95 plus” and "Age-standardized" should be selected in the "Age" column; "Both", “Male”, and ”Female” should be selected in the "Sex" column; and all the years from 1990 to 2009 should be selected in the "Year" column.
